# Circulating Noncoding RNAs Have a Promising Future Acting as Novel Biomarkers for Colorectal Cancer

**DOI:** 10.1155/2019/2587109

**Published:** 2019-06-03

**Authors:** Jia-jun Wang, Xin Wang, Yong-xi Song, Jun-hua Zhao, Jing-xu Sun, Jin-xin Shi, Zhong-hua Wu, Zhen-ning Wang

**Affiliations:** Department of Surgical Oncology and General Surgery, First Hospital of China Medical University, Shenyang 110001, China

## Abstract

Colorectal cancer (CRC) is one of the most common malignant tumors worldwide, causing a large number of cancer-related deaths each year. Patients are usually diagnosed at advanced and incurable stages due to the lack of suitable screening methods for early detection. Noncoding RNAs (ncRNAs), including small and long noncoding RNAs (lncRNA), are known to have significant regulatory functions, and accumulating evidence suggests that circulating ncRNAs have potential applications as noninvasive biomarkers for diagnosing CRC, evaluating its prognosis, or predicting chemosensitivity in the general population. In this review, we summarize the origins of circulating ncRNAs and provide details of single and multiple circulating ncRNAs that might have roles as diagnostic and prognostic biomarkers in CRC. We end by discussing circulating ncRNAs that may distinguish patients with resistance to chemotherapy.

## 1. Introduction

Colorectal cancer (CRC) is one of the most common malignant tumors of the gastrointestinal tract and the third most commonly diagnosed cancer in men and the second in women worldwide [1, 2]. Therapeutic methods have improved and new techniques have been developed, but survival rates for CRC patients are still below our expectations as they are usually diagnosed at an advanced stage [3]. Therefore, population-based early screening for CRC detection might help reduce incidence and improve patient survival [4]. Colonoscopy is the current gold standard for CRC detection, but it is not very suitable for population-wide CRC screening because it is invasive and expensive and capacity requirements cannot be met [5, 6].

Hence, it would be useful to discover novel and accurate biomarkers for screening CRC using a less invasive procedure. Recently, blood-based biomarkers such as circulating noncoding RNAs (ncRNAs) have been the subject of intense research since blood samples are easier to retrieve and more acceptable than colonoscopy for patients.

ncRNAs include microRNAs (miRNAs), long noncoding RNAs (lncRNAs), piwi-interacting RNAs (piRNAs), and transfer RNAs (tRNAs), all with no capacity to encode proteins [7–10]. In particular, miRNAs and lncRNAs have been the most widely studied ncRNAs in recent decades. miRNAs are small ncRNAs with approximately 22 nucleotides that can regulate human genes by binding to the 3′ untranslated region of the target message RNAs [11, 12]. lncRNAs, comprising more than 200 nucleotides, are involved in a wide range of biological processes and diseases including cancer development and metastasis, even though they lack an open reading frame [13–17]. Both miRNAs and lncRNAs can be detected in plasma or serum samples, and they may potentially act as circulating biomarkers for diagnosis, prognosis, and chemosensitivity in various types of cancer. For example, miR-21 was significantly upregulated in pancreatic ductal adenocarcinoma (PDAC) plasma samples compared with healthy controls. The expression of plasma miR-21 was associated with advanced stage, lymph node metastasis, liver metastasis (LM), and poor survival in PDAC patients; patients with higher plasma miR-21 have worse outcome [18]. Additionally, H19 is a well-known lncRNA found upregulated in the plasma of gastric cancer patients. H19 levels were also reduced in postoperative samples compared with preoperative samples [19]. Moreover, a panel of five miRNAs (miR-20a, miR-130, miR-145, miR-216, and miR-372) might be potential serum biomarkers for predicting the response to oxaliplatin-based chemotherapy [20].

In this review, we summarize the origins of circulating ncRNAs and discuss the current knowledge regarding their potential roles as novel diagnostic, prognostic, and chemosensitive predictive biomarkers, which may improve the effectiveness of treatments and reduce patient mortality.

## 2. Origins of Circulating ncRNAs

Most studies indicate that ncRNAs are released into the circulation via three possible mechanisms:

1. Membrane-bound vesicles such as exosomes and microvesicles are the major origin of circulating ncRNAs. These vesicles can participate in cell-cell communication by transferring ncRNAs [21–24]. Exosomes and microvesicles can carry several types of ncRNAs when released from donor cells via membrane blebbing [21, 23, 25]. Studies have shown that exosomes can transfer miRNAs to target cells and protect miRNAs from RNases in the circulation [24]. When these vesicles are received by recipient cells, the ncRNAs can participate in modulating cellular functions, such as angiogenesis, hematopoiesis, exocytosis, and tumorigenesis [21].
2. Apoptotic bodies can also be the source of circulating ncRNAs. When apoptosis occurs, cell fragments from dying cells are transported in apoptotic bodies, which are engulfed by neighboring living cells via phosphatidylserine signaling. Several miRNAs are carried within the apoptotic bodies when they are released into the circulation [26, 27]. In particular, miR-126 is highly enriched in apoptotic bodies, so uptake of apoptotic bodies by recipient cells can cause transfer of miR-126 which then regulates sprouty-related protein 1, vascular cell adhesion molecule-1, and CXCL12 [26].
3. RNA-binding proteins (RBPs) can regulate gene expression and are another possible source of circulating ncRNAs. RBPs participate in several components of the messenger RNA (mRNA) maturation process, including pre-mRNA splicing and mRNA export, localization, and translation [28, 29]. Some of the proteins that bind with ncRNAs include high-density lipoproteins (HDLs) and Argonaute 2 (Ago2) [30, 31]. Studies indicate that HDL complexes can transport miRNAs and deliver them to target cells with functional capabilities [30]. Circulating miRNAs such as miR-16, miR-92a, and miR-122 are also present in nonvesicular Ago2 complexes, and it has been suggested that these complexes are responsible for the stability of circulating miRNAs [31].

Research indicates that ncRNAs exhibit high stability in the circulation. Due to their protection in exosomes, microvesicles, apoptotic bodies, and protein complexes, circulating ncRNAs are resistant to harsh conditions such as high temperatures, extremes pH values, or long-term frozen storage [31–35]. Thus, circulating ncRNA concentrations are stable, allowing them to serve as potential biomarkers for several diseases, including CRC [33, 36, 37].

## 3. Circulating ncRNAs for Diagnostic and Early Screening in CRC

### 3.1. Single Circulating miRNAs as Diagnostic and Early Screening Biomarkers

miR-21 acts as an oncogene in several cancers [38–40], and a clear upregulation of miR-21 was found in CRC plasma [41, 42]. In a training set comprising 30 CRC patients and 30 healthy controls, the area under the receive operating characteristic (ROC) curve (AUC) value for miR-21 was 0.820 (sensitivity: 90.0%, specificity: 90.0%). In a test set containing 20 CRC patients and 20 healthy controls, the AUC value was 0.910 [41]. This association was supported by Liu et al. who found increased miR-21 in serum from CRC patients compared with colorectal advanced adenoma (CAA) patients and healthy controls, yielding an AUC value of 0.802 with a sensitivity of 65.0% and specificity of 85.0% [43]. Moreover, serum exosomal miR-21 levels could also be used for screening early CRC [44].

A group of 353 individuals (111 CRC patients, 29 inflammatory bowel disease (IBD) patients, 83 patients with benign lesions, and 130 healthy controls) participated in a study where three miRNAs (miR-24, miR-320a, and miR-423-5p) were measured, and all were decreased significantly in CRC plasma samples compared with IBD patients and controls. When miR-24, miR-320a, and miR-423-5p were used to distinguish CRC from controls, the AUC values were 0.822, 0.897, and 0.839, respectively. When these miRNAs were employed to distinguish between CRC and IBD, the AUC values for miR-24 and miR-320a were 0.974 and 0.990, respectively. Furthermore, miR-320a and miR-423-5p both decreased during the progression of colorectal disease from IBD to CRC [45].

Another study also found that circulating miRNAs could separate malignant and benign diseases from healthy controls. In a cohort of 90 CRC patients, 43 CAA patients, and 58 controls, plasma miR-760 and miR-601 levels could differentiate CRC patients from healthy controls with AUC values of 0.788 and 0.747, respectively. The AUC values were 0.682 for miR-760 and 0.638 for miR-601 when discriminating CAA patients from healthy controls. Importantly, both miRNAs decreased in the plasma during CRC progression. Patients with TNM stage IV had significantly lower plasma levels of miR-760 and miR-601 than those with stage I. In addition, ROC curve analysis showed that combining miR-601 and miR-760 with CEA improved diagnostic sensitivity from 29.4% to 80.4% with an AUC of 0.805 [36].

Other circulating miRNAs and their diagnostic value for CRC are listed in Table 1.

### 3.2. Single Circulating lncRNAs as Diagnostic and Early Screening Biomarkers

Colon cancer-associated transcript 2 (CCAT2) is located at the 8q24 region, and its genomic locus encompasses the SNP rs6983267 which is closely associated with increased risks for many cancers [46, 47]. CCAT2 is overexpressed in many cancer tissues, and it participates in tumor cell proliferation, invasion, and motility [48–50]. Compared with microsatellite-unstable CRC tissues or normal mucosae which lack the chromosomal instability, the expression level of CCAT2 is higher in microsatellite-stable CRC tissues which exhibit chromosomal instability. In addition, CCAT2 can regulate Wnt signaling via the TCF7L2 protein and also regulates the nearby gene MYC via *cis* signaling [51]. Wang et al. found higher circulating CCAT2 in CRC patient serum and exosomes than in healthy subjects. CCAT2 might be protected by exosomes and act as a novel diagnostic biomarker for predicting CRC [52].

HNF1A-AS1 was shown to be upregulated in various cancers including gastric [53], lung [54], and hepatocellular cancers [55]. The expression of HIF1A-AS1 in serum samples from 151 patients with CRC was higher than in samples from 160 healthy individuals. The diagnostic value was very high at 0.960 (sensitivity: 86.8%, specificity: 92.5%). In addition, serum HIF1A-AS1 levels were strongly associated with differentiation degree, tumor size, T stage, N stage, M stage, and TNM stage [56].

NEAT1 was shown to be overexpressed in CRC serum and cancer tissues compared with healthy controls and matched NATs. ROC curve analysis indicated the discriminatory power of NEAT1 levels in tissues with an AUC value of 0.810 [39]. In serum samples, NEAT1 was significantly elevated in 56 CRC patients compared with controls, and the AUC value was 0.947 [57]. Considering the diagnostic relevance of NEAT1, future studies should expand the sample size to hundreds of individuals in further multicenter studies for possible clinical applications.

Colorectal Neoplasia Differentially Expressed (CRNDE), which was originally found aberrantly expressed in CRC, is upregulated in a number of malignant cancers such as pancreatic, lung, and hepatocellular cancers [58–60]. It can promote cell proliferation and chemoresistance by regulating Wnt/*β*-catenin signaling via miR-181a-5p in CRC [61]. CRNDE is located at human chromosome 16 and many splice variants have been identified, one of which called CRNDE-h was shown to effectively distinguish between colorectal malignancies, benign diseases, and healthy individuals. Serum exosomal CRNDE-h levels were significantly upregulated in CRC patients compared with patients with IBD, hyperplastic polyps, adenoma, or healthy controls. The AUC value was 0.892 for distinguishing CRC patients from a group containing 80 benign disease patients and 80 controls (sensitivity: 70.3%, specificity: 94.4%). The diagnostic value of CRNDE-h was better than that of the conventional tumor biomarker CEA, which alone had an AUC value of 0.688 (sensitivity: 37.16%, specificity: 88.75%). The AUC value improved significantly to 0.913 when exosomal CRNDE-h levels were combined with CEA. The origin of exosomal CRNDE-h has been explored. It was shown that exosomal CRNDE-h could enter the cell culture medium and that expression was clearly elevated in five CRC cell lines (HCT116, SW620, SW480, HT29, and LoVo). Second, the presence of a tumor led to a marked increase in the serum exosomal CRNDE-h level in a xenograft mice model. Third, CRNDE-h expression levels measured in serum samples and matched CRC tissues showed a moderately significant correlation. Finally, serum exosomal CRNDE-h levels were significantly lower in postoperative samples compared with preoperative samples. These findings suggest that the exosomal CRNDE-h detected in the serum is mainly released or leaked from tumor cells. Thus, exosomal CRNDE-h may be a novel serum-based tumor marker for the diagnosis of CRC [62]. Besides CRNDE-h, another splice variant of CRNDE named CRNDE-p might also be a diagnostic biomarker. Yu et al. indicated that serum exosomal CRNDE-p from 410 CRC patients was higher than that in 58 adenoma patients or 175 healthy subjects. The AUC for CRNDE-p discriminating CRC patients from adenoma patients is 0.854, and the AUC is 0.882 when combining serum exosomal CRNDE-p and the traditional biomarker CEA. In addition, high expression of CRNDE-p is closely associated with advanced T stage lymph node metastasis and clinical stages. This suggests that serum exosomal CRNDE-p might be a novel diagnostic biomarker, especially when combined with the traditional biomarker CEA [63].

Hu et al. isolated plasma exosomes by ultracentrifugation from 10 CRC patients and 10 healthy individuals then used microarray to find lncRNAs with differential expression. Among the 1705 significantly differential lncRNAs, they chose the six lncRNAs with the largest increase in expression (LNCV6_116109, LNCV6_98390, LNCV6_38772, LNCV6_108266, LNCV6_84003, and LNCV6_98602) for subsequent analysis. In a larger cohort consisting of 50 CRC patients and 50 healthy subjects, researchers found that the expression levels of all six lncRNAs are significantly higher in CRC than in healthy individuals. These six plasma exosomal lncRNAs might serve as potential biomarkers for early CRC detection. All six lncRNAs are obviously higher in CRC patients with stage I/II than in healthy subjects. AUC values for LNCV6_116109, LNCV6_98390, LNCV6_38772, LNCV6_108266, LNCV6_84003, and LNCV6_98602 are 0.8052, 0.7088, 0.7460, 0.7292, 0.7356, and 0.6800, respectively [64].

ZNFX1 antisense RNA1 (ZFAS1) has been reported to be overexpressed and involved in cell proliferation and metastasis in many cancers [65–67]. Additionally, studies demonstrate that SP-1 can induce ZFAS1 and promote cell cycle progression via the miR-150-5p/VEGFA axis [68]. ZFAS1 also acts as an oncogene by destabilizing p53 and its interactions with the CDK1/cyclin B complex, finally regulating the cell cycle and inhibiting apoptosis in CRC [69]. Fang et al. examined expression levels of ZFAS1 in plasma samples from 105 patients with CRC and 95 healthy subjects and found that ZFAS1 is higher in plasma samples from CRC patients, similar to its change in tissues. When the optimal cutoff value is 10.84, ZFAS1 has an AUC of 0.88 and sensitivity and specificity of 92.38% and 76.84%, respectively. Moreover, its positive predictive value and negative predictive value are 80.70% and 84.88%, respectively [70]. Thus, ZFAS1 shows potential as a diagnostic biomarker.

HOTAIRM1 is located between the human HOXA1 and HOXA2 genes, and its level was shown to be lower in CRC plasma samples compared with controls. In a training set of 100 CRC patients and 67 controls, the AUC value was 0.780 (specificity: 80.3%, sensitivity: 61.5%). In the validation set comprising 50 CRC patients and 34 controls, the AUC value was 0.771 (specificity: 76.5%, sensitivity: 64.0%) [71].

GNAT1-1 was found downregulated in CRC tissues and plasma samples compared with matched NATs and healthy controls. Lower GNAT1-1 expression was associated with more advanced stages, and patients with TNM stages III and IV have significantly lower plasma GNAT1-1 levels than those with stages I and II. Moreover, GNAT1-1 could discriminate CRC patients from controls with an AUC value of 0.720 [72].

GAS5 is downregulated in CRC tissues compared with matched NATs. Some previous results indicated that GAS5 can inhibit CRC progression via the miR-182-5p/FOXO3a axis and the Wnt/*β*-catenin signaling pathway [73–75]. In a recent study, Liu et al. found that GAS5 is downregulated in CRC tissues, plasma, and exosomes, with an AUC for tissue GAS5 levels distinguishing CRC and NATs of 0.791 and GAS5 in plasma and exosomes distinguishing 158 patients with CRC and 173 healthy subjects with AUC values of 0.875 and 0.964, respectively [76]. Further research confirms this result, demonstrating an obvious decrease in GAS5 levels in the serum of CRC patients between 109 CRC patients and 99 healthy subjects [77]. These results suggest that GAS5 might be intimately involved in CRC and acts as a biomarker for CRC screening.

Other circulating lncRNAs and their diagnostic values are listed in Table 1.

### 3.3. Panels of Circulating miRNAs and lncRNAs as Diagnostic and Early Screening Biomarkers

In addition to the single circulating ncRNAs reviewed above, studies have also combined different circulating ncRNAs into panels for detecting CRC. Combinations of several ncRNAs have better diagnostic value than single biomarkers [78]. Based on a cohort comprising control, CRC, CAA, breast cancer, pancreatic cancer, and lung cancer samples, Carter et al. identified some uniquely dysregulated miRNAs specifically for screening CRC. Four miRNAs (miR-21, miR-29c, miR-346, and miR-374a) could distinguish between healthy controls or patients with any type of neoplasia with an AUC of 0.91. Four miRNAs (miR-21, miR-29c, miR-372, and miR-374a) were analyzed in patients with neoplasms, and the AUC value was 0.79 when discriminating patients with colorectal neoplasm from other neoplasms. Subsequently, miR-29c, miR-122, miR-192, and miR-374a were used to distinguish whether patients had CRC or CAA, with an AUC value of 0.98 [79].

Considering the diagnostic relevance of this miRNA signature, further studies are merited before clinical applications. In another study, four plasma miRNAs (miR-21, miR-25, miR-18a, and miR-22) were identified as CRC biomarkers. The combination of these four miRNAs could clearly distinguish CRC patients from controls, with an AUC value of 0.93 (sensitivity: 67%, specificity: 90%) [80]. A microRNA expression profiling assay was used for screening biomarkers to distinguish CRC, CRC precursor lesions, and healthy individuals. Total RNA was obtained from 21 patients with CRC, 20 patients with CAA, and 20 healthy controls for microRNA profiling. Six miRNAs (miR-29a, miR-18a, miR-19a, miR-19b, miR-15b, and miR-335) were significantly upregulated in CRC plasma samples compared with healthy controls. Combining miR-19a and miR-19b showed an AUC value of 0.8194, with sensitivity and specificity of 78.57% and 77.36%, respectively. Combining miR-15b with these two miRNAs increased the discriminative capacity, with an AUC value of 0.8356 (sensitivity: 78.6%, specificity: 79.3%) [78].

Wang et al. combined up- and downregulated miRNAs and established a diagnostic panel for CRC screening. miR-21 and let-7g were both upregulated in CRC serum samples, whereas miR-92a, miR-31, miR-181b, and miR-203 were all downregulated. In a training set comprising 30 CRC patients and 30 healthy controls, this panel of six miRNAs yielded an AUC value of 0.900. Subsequent validation obtained an AUC value of 0.923 when distinguishing 83 CRC patients and 59 controls [81].

Plasma expression levels of HOTAIR and CCAT1 were found to be remarkably upregulated in CRC patients compared with healthy individuals. Combining these two lncRNAs increased diagnostic performance, with an AUC value of 0.954 (sensitivity, 84.3%; specificity, 80.2%). Additionally, the diagnostic positivity rate when combining HOTAIR with CCAT1 for CRC in stage I/II was 85% [82].

Dysregulated lncRNAs were investigated in CRC tissues using genome-wide lncRNA microarrays, and their expression levels were then validated in 80 cancer tissues and 120 serum samples. A panel of four lncRNAs (lnc-BANCR, lnc-NR-026817, lnc-NR-029373, and lnc-NR-034119) obtained an AUC value of 0.881 when discriminating CRC patients and controls (sensitivity: 89.2%, specificity: 75.8%). The corresponding AUC values obtained using this panel for CRC patients with TNM at stage I, stage II, and stage III were 0.774, 0.844, and 0.949, respectively [35].

In another study, Wang et al. found that a three-lncRNA signature could play as a diagnostic marker for CRC screening via stepwise regression analysis. First, they found that 13 of the 17 candidate CRC or gastrointestinal cancer-associated lncRNAs were detectable in a small cohort. Second, five of the 13 lncRNAs were found with significant differential abundance in 30 preoperative CRC patients and 31 healthy individuals. Third, these five lncRNAs were further evaluated in additional serum samples from 30 CRC patients and 30 healthy individuals. Finally, all data from the second and third steps were pooled and analyzed, with results indicating that RP11-462C24.1, LOC285194, and Nbla12061 were significantly upregulated in serum from CRC patients. The AUC value of combining RP11-462C24.1, LOC285194, and Nbla12061 was 0.793 (sensitivity: 68.3%, specificity: 86.9%), obviously higher than that of CEA, CA199, CA125, and CA724. When these three lncRNAs were combined with CEA, CA199, CA125, or CA724, the AUC values further improved to 0.845, 0.855, 0.798, or 0.824, respectively. Furthermore, expression of the three lncRNAs was significantly reduced after surgery. These results suggest that this combination of three lncRNAs in serum represents a new supplementary method for CRC screening [37]. Panels of circulating miRNAs and lncRNAs that act as diagnostic biomarkers are listed in Table 2.

## 4. Circulating ncRNA as Recurrence and Survival Evaluation Biomarkers in CRC

### 4.1. Single Circulating miRNAs as Recurrence and Survival Evaluation Biomarkers

Increased serum miR-21 strongly correlated with poor survival in CRC patients, and it might serve as an independent prognostic factor for overall survival (OS). Furthermore, elevated miR-21 expression in serum samples correlated with tumor size and distant metastases [83]. The same result was obtained by Yin et al. who found elevated serum miR-21 levels in patients with LM and other organ metastasis [84]. Similarly, another study showed that increased exosomal miR-21 in CRC plasma samples significantly correlated with advanced TNM stage and LM. Patients with high levels of exosomal miR-21 had poor OS and relapse-free survival (RFS). Furthermore, plasma exosomal miR-21 levels could serve as an independent prognostic factor for OS and disease-free survival (DFS) in TNM stage II and III patients, and OS in TNM stage IV patients [85]. miR-200c was significantly elevated in TNM stage IV serum samples compared with TNM stage I. High serum miR-200c levels were significantly associated with poor OS, DFS, positive lymph nodes, and LM. miR-200c could serve as an independent prognostic factor for lymph node metastasis, tumor recurrence, and poor OS in CRC patients [86].

Similarly, Hur et al. indicated that miR-885-5p was a significantly upregulated miRNA in the LM group compared with the pCRC group. miR-885-5p expression levels significantly correlated with lymph node metastasis, distant metastasis, and LM. Furthermore, patients with higher serum miR-885-5p expression levels had poor OS and DFS [87]. miR-885-5p might serve as a potential biomarker for CRC prognosis. Yuan et al. demonstrated that miR-183 was significantly overexpressed in CRC plasma samples, and patients with elevated expression levels of miR-183 had a high risk of tumor recurrence. In addition, miR-183 expression could serve as an independent prognostic factor for OS in CRC patients. High plasma miR-183 levels were significantly associated with lymph node metastasis, distant metastasis, and advanced pTNM stage [88].

Another study showed that miR-139-5p might be a CRC recurrence-associated biomarker because miR-139-5p levels were significantly higher in cancer tissues from recurrent patients. A subsequent study demonstrated that miR-139-5p levels in serum samples were significantly higher in recurrent CRC patients compared with nonrecurrence cases, with an AUC value of 0.750, a specificity of 80.0%, and a sensitivity of 64.0%. Furthermore, CRC patients with higher serum levels of miR-139-5p had a significantly shorter RFS than those with lower miR-139-5p expression [89].

Other circulating miRNAs are listed in Table 3, which shows their prognostic value for CRC.

### 4.2. Single Circulating lncRNAs as Recurrence and Survival Evaluation Biomarkers

lncRNA 91H is an oncogene involved with CRC progression, and it can promote cell proliferation, migration, and invasion [90]. Serum exosomal 91H levels strongly correlate with metastasis and tumor recurrence. Patients with high 91H levels had a higher risk of tumor metastasis and recurrence than other patients. Univariate and multivariate analyses indicated that 91H could serve as an independent prognostic factor for RFS in CRC patients [91].

Increased serum exosomal CRNDE-h was significantly associated with regional lymph nodes and distant metastasis. Furthermore, patients with high exosomal CRNDE-h had poor OS, and expression of exosomal CRNDE-h could serve as an independent factor for OS in CRC patients [62].

As mentioned above, GNAT1-1 is significantly downregulated in CCRC serum samples. The expression of GNAT1-1 expression was significantly lower in LM tissues compared with pCRC tissues. In addition, GNAT1-1 strongly correlated with tumor stage, lymphovascular invasion, tumor depth, and distant metastasis. Patients with decreased GNAT1-1 expression levels have a shorter OS than those with high levels, and GNAT1-1 expression could be used as an independent prognostic factor [72].

## 5. Circulating ncRNAs as Treatment Response Prediction Biomarkers in CRC

### 5.1. Circulating miRNAs as Treatment Response Prediction Biomarkers

Chemotherapy is a useful treatment for CRC patients before or after surgical resection. Effective systemic treatment could improve the possibility of survival with advanced stage CRC. However, CRC patients with resistance to chemotherapy fail to benefit from effective chemotherapy and may also suffer from adverse side effects following chemotherapy [20, 92, 93]. To improve CRC treatment, it is necessary to identify new therapeutic biomarkers to discriminate patients who will respond to chemotherapy from those who are resistant. Recently, several studies demonstrated associations between circulating ncRNAs and sensitivity to chemotherapy. The association between ncRNAs and chemosensitivity are listed in Table 4, which shows their prognostic value for CRC.

Studies have identified potential serum biomarkers for predicting the response to oxaliplatin-based chemotherapy (modified FOLFOX6) in patients with CRC. In particular, a study employed TaqMan low-density arrays based on pooled serum samples from 20 responders and 20 nonresponders to chemotherapy to identify differentially expressed miRNAs. The results showed that five serum miRNAs (miR-20a, miR-130, miR-145, miR-216, and miR-372) differed significantly between the two groups. In the training set, the AUC value for these five miRNAs was 0.841, and the positive and negative predictive values were 0.86 and 0.89, respectively. Moreover, in a larger validation set comprising 93 responders and 80 nonresponders, the AUC value was 0.918, and the positive and negative predictive values were 0.93 and 0.94, respectively. These five serum miRNAs may be potential serum biomarkers for predicting response to chemotherapy in CRC [20].

Another study identified potential biomarkers for predicting outcome in metastatic CRC (mCRC) patients treated with 5-FU and oxaliplatin-based chemotherapy. A cohort comprising 24 mCRC plasma samples (12 responders and 12 nonresponders) was investigated in this study. The top 10 differentially expressed miRNAs were selected for further study in a validation cohort of 150 patients, and three plasma miRNAs (miR-106a, miR-484, and miR-130) were found to be significantly upregulated in mCRC patients with chemotherapy resistance. Patients with elevated expression of these three plasma miRNAs had poor progression-free survival (PFS). These plasma miRNAs might predict the outcome for mCRC patients before treatment with 5-FU and oxaliplatin-based chemotherapy [94].

Similarly, Hansen et al. found significantly elevated plasma miR-126 levels in nonresponding mCRC patients compared with responding patients who received first-line chemotherapy (XELOX) combined with bevacizumab. The change in miRNA-126 also positively correlated with tumor size changes. Thus, there is a relationship between changes in miR-126 and tumor response when receiving first-line chemotherapy combined with bevacizumab in mCRC patients. Therefore, miR-126 might be a possible biomarker for resistance to antiangiogenic-containing treatments [95].

miR-1914 ∗ and miR-1915 were found to be downregulated in the plasma of chemoresistant CRC patients who received XELOX. The decreased expression of these two miRNAs significantly correlated with poor OS and PFS. miR-1914 ∗ and miR-1915 can reduce the expression of nuclear factor I/X and suppress chemoresistance by regulating cell proliferation, invasion, and apoptosis in CRC [96].

### 5.2. Circulating lncRNAs as Treatment Response Prediction Biomarkers

lncRNA XIST was shown to be significantly upregulated in both serum and cancer tissues from nonresponding CRC patients. Serum XIST had an AUC value of 0.756 when distinguishing nonresponding cases from responders (sensitivity: 71.7%, specificity: 68.3%). Furthermore, OS and RFS were poor in CRC patients with elevated XIST expression levels who were treated with 5-FU [97].

Serum lncRNA MEG3 levels were significantly lower in oxaliplatin-based chemotherapy resistant CRC patients, with an AUC value of 0.784 when discriminating nonresponders from responders [98].

## 6. Future Directions

CRC is a leading cause of cancer-related deaths throughout the world [99]. Colonoscopy is the gold standard for diagnosis, but it is expensive and invasive [5, 6]. Thus, novel and accurate biomarkers using less invasive approaches are urgently required to improve CRC detection. In recent decades, studies have shown that ncRNAs can be detected in various bodily fluids, including serum and plasma, and that they are particularly stable [24, 31–34]. Significant progress has been made in investigating the potential roles of ncRNAs in CRC screening.

Several traditional blood-based biomarkers, such as CEA, carbohydrate antigen (CA) 19-9, CA242, and CA724, have been employed widely as clinical diagnostic biomarkers for CRC screening in recent decades, but they are limited in their diagnostic value, sensitivity, and specificity. As discussed above, several circulating ncRNAs have high accuracy in CRC detection, such as CRNDE-h with an AUC value of 0.892, a sensitivity of 70.3%, and specificity of 94.4% when differentiating CRC and controls, and they may potentially be more reliable biomarkers compared with traditional blood-based biomarkers (in the same cohort, the AUC value for CEA was only 0.688 with a sensitivity of 37.16% and a specificity of 88.75%; combining CRNDE-h and CEA improved the AUC value from 0.688 to 0.913) [62]. Also, CEA protein expression levels may be affected by other bowel diseases, such as ulcerative colitis [100]. Some other malignant tumors, including PDAC, breast cancer, and gastric cancer, can also affect levels of these traditional markers [100–102], leading to frequent high false positive rates. This may be avoided using ncRNAs, as Carter et al. found that miR-21, miR-29c, miR-372, and miR-374a could distinguish CRC and other neoplasms with an AUC value of 0.79. miR-29c, miR-122, miR-192, and miR-374a could also distinguish CRC from CAA, with an AUC value of 0.98 [79]. Circulating ncRNAs have remarkable potential as biomarkers for CRC screening. In the future, it will be important to identify further circulating ncRNA biomarkers, possibly in combination with each other or protein biomarkers, and apply them in the clinic.

Many recent studies have focused on lncRNAs and miRNAs, but few have investigated other types of circulating ncRNAs such as circular RNAs (circRNAs) and piRNAs. circRNAs can regulate genome expression levels by acting as miRNA sponges, and they are extremely stable since they lack open linear tails and they are insensitive to exonucleases [103–105]. Yang et al. found upregulated circ-LDLRAD3 in the plasma of patients with pancreatic cancer that could be used as a biomarker in diagnosing pancreatic cancer [106]. The potential use of serum piRNA was also suggested as a diagnostic biomarker for tumor detection. piR-651 was significantly downregulated in classical Hodgkin lymphoma serum samples, and it exhibited an increasing trend in serum samples from complete response patients compared with the diagnostic samples [107]. However, few studies have investigated the diagnostic capacities of circulating circRNAs and piRNAs for CRC detection. Among the human genome, dozens or even hundreds of genes or transcripts may serve as accurate and sensitive biomarkers.

The studies summarized above suggest areas for further improvements. Circulating ncRNAs were often evaluated in small cohorts of serum and plasma samples in these studies. For example, Kanaan et al. investigated miR-21 in a group of 20 CRC patients and 20 healthy subjects, and the AUC value was 0.910 [41], yet this high diagnostic efficacy might accidentally be misattributed due to the small sample size. In future investigations, multicenter studies should be performed and sample sizes must be increased to ensure reliable scientific results.

Some studies have combined multiple circulating ncRNAs into panels for CRC detection. These panels obtained higher AUC values and improved the diagnostic accuracy for CRC detection over most single biomarkers [78]. Exosomal miR-23a and miR-1246 levels were validated in 101 CRC serum samples, and AUC values of 0.953 and 0.948 were obtained, respectively [44]. Considering the high predictive accuracy of these individual circulating miRNAs, combining serum exosomal miR-23a and miR-1246 levels may yield a higher AUC value. Establishing mathematical models and combining multiple biomarkers may be an effective approach for optimizing diagnostic biomarkers. We hypothesize that combining different types of ncRNAs, such as lncRNAs and miRNAs which have known diagnostic capacities, may yield novel and more precise biomarker panels in future research.

Several studies have reported the potential roles of circulating ncRNAs as diagnostic, prognostic, or chemosensitivity predictive biomarkers in CRC, but they may be “the tip of the iceberg.” Future research should aim at achieving a deeper understanding of the regulatory mechanisms related to circulating ncRNAs and establish standard protocols for ncRNA detection to establish them as biomarkers for general CRC patients.

## Figures and Tables

**Table 1:** Single circulating ncRNA as diagnostic and early screening biomarkers for CRC.

ncRNAs	Body fluid	Dysregulation	Numbers of CRC	Numbers of healthy control	AUC	Sensitivity	Specificity	Reference
miR-21	Plasma	↑	50	50	0.91	90%	90%	[41]
	Serum	↑	200	80	0.802	65%	85%	[43]
miR-92a	Serum	↑	200	80	0.786	65.5%	82.5%	[43]
	Plasma	↑	120	115	0.885	89%	70%	[108]
	Plasma	↑	120	59	0.838	84.0%	71.2%	[109]
miR-29a	Plasma	↑	120	59	0.844	69.0%	89.1%	[109]
miR-18a	Plasma	↑	78	86	0.804	73.1%	79.1%	[110]
miR-200c	Plasma	↑	78	86	0.749	64.1%	73.3%	[110]
miR-20a	Plasma	↑	100	79	0.59	46.00%	73.00%	[111]
miR-106a	Plasma	↑	100	79	0.605	74.00%	44.40%	[111]
miR-199a-3p	Serum	↑	114	32	0.644	47.60%	75.00%	[112]
miR-223	Serum	↑	130	60	0.838	—	—	[113]
miR-372	Serum	↑	165	30	0.854	81.9%	73.3%	[114]
miR-103	Serum	↑	124	32	0.662	55.9%	75.0%	[115]
miR-720	Serum	↑	124	32	0.63	58.3%	56.3%	[115]
miR-155	Serum	↑	146	60	0.776	58.2%	95.0%	[116]
miR-378	Plasma	↑	65	70	0.796	—	—	[117]
miR-23a	Serum (exosome)	↑	101	19	0.953	—	—	[44]
miR-150	Serum (exosome)	↑	101	19	0.758	—	—	[44]
miR-223	Serum (exosome)	↑	101	19	0.716	—	—	[44]
miR-1246	Serum (exosome)	↑	101	19	0.948	—	—	[44]
miR-221	Plasma	↑	103	37	0.606	86.00%	41.00%	[118]
miR-24	Plasma	↓	111	130	0.839	78.38%	83.85%	[45]
miR-320a	Plasma	↓	111	130	0.886	92.79%	73.08%	[45]
miR-423-5p	Plasma	↓	111	130	0.833	91.89%	70.77%	[45]
miR-601	Plasma	↓	100	68	0.747	69.2%	72.4%	[36]
miR-760	Plasma	↓	100	68	0.788	80.0%	72.4%	[36]
miR-194	Serum	↓	55	55	0.85	72%	80%	[119]
miR-29b	Serum	↓	55	55	0.87	77%	75%	[119]
miR-139-3p	Serum	↓	117	90	0.9935	96.60%	97.80%	[120]
miR-375	Plasma	↓	94	46	0.7489	76.92%	64.62%	[121]
miR-145	Serum	↓	25	10	0.78	80%	68%	[122]
HIF1A-AS1	Serum	↑	151	160	0.96	86.80%	92.5%	[56]
CRNDE-h	Serum (exosome)	↑	148	300	0.892	70.3%	94.4%	[62]
CRNDE-p	Serum (exosome)	↑	410	175	0.854	0.854	—	[63]
HOTAIRM1	Plasma	↓	150	101	0.78	64.0%	76.5%	[71]
ZFAS1	Plasma	↑	105	95	0.88	92.38%	76.84%	[70]
GNAT1-1	Plasma	↓	62	37	0.72	—	—	[72]
BLACAT1	Serum	↑	30	30	0.858	83.3%	76.7%	[123]
CCAT2	Serum (exosome)	↑	100	—	—	—	—	[52]
GAS5	Plasma (exosome)	↓	158	173	0.875	—	—	[76]
LNCV6_116109	Plasma (exosome)	↑	50	50	0.8052	—	—	[64]
LNCV6_98390	Plasma (exosome)	↑	50	50	0.7088	—	—	[64]
LNCV6_38772	Plasma (exosome)	↑	50	50	0.7460	—	—	[64]
LNCV6_108266	Plasma (exosome)	↑	50	50	0.7292	—	—	[64]
LNCV6_84003	Plasma (exosome)	↑	50	50	0.7356	—	—	[64]
LNCV6_98602	Plasma (exosome)	↑	50	50	0.6800	—	—	[64]

Note: ↑, upregulated; ↓, downregulated; —, not mentioned. HIF1A-AS1, hypoxia-inducible factor 1 alpha-antisense RNA 1; CRNDE, colorectal neoplasia differentially expressed; HOTAIRM1, HOX antisense intergenic RNA myeloid 1; GNAT1-1, G protein subunit *α* transducin 1; BLACAT1, bladder cancer-associated transcript 1; ZFAS1, ZNFX1 antisense RNA1; GAS5, growth arrest specific transcript 5; CCAT2, colon cancer-associated transcript 2.

**Table 2:** Panels of circulating miRNAs as diagnostic and early screening biomarkers for CRC.

Panel of ncRNA	Body fluid	Number of ncRNAs	Numbers of patients	Numbers of controls	AUC	Sensitivity	Specificity	Reference
miR-21↑, let-7g↑, miR-31↓, miR-92a↓, miR-181b↓, miR-203↓	Serum	6	113	89	0.923	—	—	[81]
miR-19a↑, miR-19b↑, miR-15b↑	Plasma	3	63	73	0.84	78.57%	79.25%	[78]
miR-409-3p↑, miR-7↓, miR-93↓	Plasma	3	124	117	0.897	82%	89%	[32]
miR-29c↓, miR-122↑, miR-192↓, miR-374a↓	Plasma	4	55	55	0.98 (discriminate CRC from CAA)	—	—	[79]
lnc-CCAT1↑, lnc-HOTAIR↑	Plasma	2	32	32	0.954	—	—	[82]
lnc-LOC285194↑, lnc-RP11-462C24.1↑, lnc-Nbla12061↑	Serum	3	71	70	0.793	68.33%	86.89%	[37]
lnc-BANCR↑, lnc-NR-026817↓, lnc-NR-029373↓, lnc-NR-034119↓	Serum	4	240	240	0.881	89.17%	75.83%	[35]

Note: ↑, upregulated; ↓, downregulated; —, not mentioned. CAA, colorectal advanced adenoma; CCAT1, colon cancer-associated transcript 1; HOTAIR, HOX transcript antisense intergenic RNA; BANCR, BRAF-activated noncoding RNA.

**Table 3:** Single circulating ncRNAs as recurrence and survival evaluation biomarkers for CRC.

ncRNAs	Body fluid	Numbers of patients	Clinical significance	Application detail	Reference
miR-21	Serum	186	Tumor size, distant metastasis	miR-21↑ → OS↓	[83]
miR-22	Plasma (exosome)	326	Liver metastasis, TNM stage	miR-21↑ → OS↓, DFS↓	[85]
miR-200c	Serum	206	Lymph node, distant metastasis, tumor recurrence	miR-200c↑ → OS↓, DFS↓	[86]
miR-96	Plasma	227	—	miR-96↑ → OS↓	[124]
miR-200b	Plasma	227	—	miR-200b↑ → OS↓	[124]
miR-141	Plasma	227	—	miR-141↑ → OS↓	[124]
miR-885-5p	Serum	169	Lymph node metastasis, distant metastasis, TNM stage, liver metastasis, lymphatic invasion	miR-885-5p↑ → OS↓, DFS↓	[87]
miR-221	Serum	103	—	miR-221↑ → OS↓	[118]
miR-183	Plasma	118	Lymph node metastasis, distant metastasis, pTNM stage (III-IV), tumor recurrence	miR-183↑ → OS↓, DFS↓	[88]
miR-139-5p	Serum	76	TNM stage, tumor recurrence	miR-139-5p↑ → RFS↓	[89]
miR-148a	Serum	26	Tumor recurrence	miR-148a↓ → OS↓, DFS↓	[125]
91H	Serum (exosome)	232	Tumor metastasis, recurrence	91H↑ → RFS↓	[91]
CRNDE-h	Serum (exosome)	148	Lymph nodes metastasis, distant metastasis	CRNDE-h↑ → OS↓	[62]
GNAT1-1	Plasma	62	—	GNAT1-1↓ → OS↓	[72]

Note: ↑, upregulated; ↓, downregulated; —, not mentioned. OS, overall survival; DFS, disease-free survival; RFS, relapse-free survival; CRNDE-h, colorectal neoplasia differentially expressed-h; SPRY4-IT1, SPRY4 intronic transcript 1.

**Table 4:** Single circulating ncRNAs as treatment response prediction biomarkers for CRC.

Chemotherapy regimen	ncRNA	Body fluid	Chemotherapy sensitivity	Reference
5-Fluorouracil combined with oxaliplatin	miR-106a, miR-484, miR-130	Plasma	(miR-106a, miR-484, miR-130) ↑ → resistance	[94]
	miR-20a, miR-130, miR-145, miR-216, miR-372	Serum	(miR-20a, miR-130, miR-145, miR-216, miR-372) ↑ → resistance	[20]
Oxaliplatin combined with capecitabine	miR-1914, miR-1915	Plasma	(miR-1914↑, miR-1915) ↑ → responding	[96]
Oxaliplatin, capecitabine combined with bevacizumab	miR-126	Plasma	miR-126↑ → resistance	[95]
5-Fluorouracil	XIST	Serum	XIXT↑ → resistance	[97]
Oxaliplatin	MEG3	Serum	MEG3↑ → resistance	[98]

Note: ↑, upregulated. XIST, X-inactive specific transcript; MMEG3, maternally expressed gene 3.
